# Supplementary material for: Exploration of drug resistance mechanisms in triple negative breast cancer cells using a microfluidic device and patient tissues
Source: eLife. 2024 Mar 27;12:RP88830. doi: 10.7554/eLife.88830 (PMC10972559; doi:10.7554/eLife.88830)
Supplement: Supplementary file 1. [file elife-88830-supp1.docx]

**Table S1. List of primer sequences.**

|  |  |  |
| --- | --- | --- |
| **ChIP assay primers** | | |
| **Gene Name** | **Forward Primer** | **Reverse Primer** |
| *NUPR1* -1400/-1000 | GATCTCAGCTCACCACAACCT | ATCTTCTTCCTAGAGTTGGGGAGA |
| *NUPR1* -1000/-600 | ACAATTATTATCATCCTTATTTTACAG | ATAGACATCTGCCACCATGCC |
| *NUPR1* -600/-200 | AATCCCAGCTATTCGGGAGG | ATATTTTCCATAGAGGAGGTCCCG |
| *NUPR1* -200/+200 | CCAGCTGGGTGAGCCTGG | AGATGGCTGAGTGGGCCTTA |
|  |  |  |
| **Real-time qPCR primers** | | |
| **Gene Name** | **Forward Primer** | **Reverse Primer** |
| *GAPDH* | GTGTTCCTACCCCCAATGTGT | ATTGTCATACCAGGAAATGAGCTT |
| *NUPR1* | TCGGGCCTCTCATCATGCCT | TGCCCCTCGCTTCTTCCTCT |
| *HDAC*1 | ATATCGGGGCTGGCAAAGGC | TCCACACACTTGGCGTGTCC |
| *HDAC*2 | AGGCCCCATAAAGCCACTGC | CTCCAGCAACTGAACCGCCA |
| *HDAC*3 | CTCAGCATCCGAGGGCATGG | TCGATGCGGGTGCTGACATC |
| *HDAC*4 | GGTGGTGTTGGGGTGGACAG | GCTCTCCTCCGCATGGTGTC |
| *HDAC*5 | CAACAGCTCCCACAGCACCA | TTGGTGACAGTGACCGTGGC |
| *HDAC*6 | GACCTTGGAGCTAGGCAGCG | TGCCACCAAATGGGGACACC |
| *HDAC*7 | CAGTGACCGCAGGACCCATC | CTGGGCAAAGTGGAAGGGCA |
| *HDAC*8 | CAACACGGCTCGATGCTGGA | CGGCAGCTTGGCGTGATTTC |
| *HDAC*9 | TATGGCACCAACCCCCTGGA | GCACCGGACGAGTGTAGCTC |
| *HDAC*10 | CCTGGCCTATGGCTTCCAGC | TGCTAGCTGGGGTGTGGAGT |
| *HDAC*11 | GTGCACACGAGGCGCTATCT | GCCAGCTTCCCCGCCATTAT |
